# Genome-wide association studies of *Striga* resistance in extra-early maturing quality protein maize inbred lines

**DOI:** 10.1093/g3journal/jkac237

**Published:** 2022-09-08

**Authors:** Gbemisola Okunlola, Baffour Badu-Apraku, Omolayo Ariyo, Paterne Agre, Queen Offernedo, Moninuola Ayo-Vaughan

**Affiliations:** Maize Improvement Programme, International Institute of Tropical Agriculture, IITA, Oyo Road, Ibadan 200001, Oyo ,5320, Nigeria; Department of Plant Breeding and Seed Technology, Federal University of Agriculture, Abeokuta 110124, Ogun, 2240, Nigeria; Maize Improvement Programme, International Institute of Tropical Agriculture, IITA, Oyo Road, Ibadan 200001, Oyo ,5320, Nigeria; Department of Plant Breeding and Seed Technology, Federal University of Agriculture, Abeokuta 110124, Ogun, 2240, Nigeria; Maize Improvement Programme, International Institute of Tropical Agriculture, IITA, Oyo Road, Ibadan 200001, Oyo ,5320, Nigeria; Maize Improvement Programme, International Institute of Tropical Agriculture, IITA, Oyo Road, Ibadan 200001, Oyo ,5320, Nigeria; Department of Plant Breeding and Seed Technology, Federal University of Agriculture, Abeokuta 110124, Ogun, 2240, Nigeria

**Keywords:** SNP markers, *Striga hermonthica*, marker-assisted selection, quality protein maize

## Abstract

Identification of genes associated with *Striga* resistance is invaluable for accelerating genetic gains in breeding for *Striga* resistance in maize. We conducted a genome-wide association study to identify genomic regions associated with grain yield and other agronomic traits under artificial *Striga* field infestation. One hundred and forty-one extra-early quality protein maize inbred lines were phenotyped for key agronomic traits. The inbred lines were also genotyped using 49,185 DArTseq markers from which 8,143 were retained for population structure analysis and genome wide-association study. Cluster analysis and population structure revealed the presence of 3 well-defined genetic groups. Using the mixed linear model, 22 SNP markers were identified to be significantly associated with grain yield, *Striga* damage at 10 weeks after planting, number of emerged *Striga* plants at 8 and 10 weeks after planting and ear aspect. The identified SNP markers would be useful for breeders for marker-assisted selection to accelerate the genetic enhancement of maize for *Striga* resistance in sub-Saharan Africa after validation.

## Introduction

*Striga hermonthica* parasitism is fast becoming an endemic in West Central Africa because of its increased dispersal mechanisms which include wind, animals, farm implements, and surface water (Ejeta 2007). The geographical distribution and the level of infestation of this parasitic weed are steadily increasing and more increase is expected because of the adverse effects of climate change (Mohamed *et al.* 2007). *Striga* lacks its own root system, and therefore depends completely on maize for nutrients and water for survival for 6–8 weeks while still under the ground once attached to the host plant (Bebawi and Mutwali 1991; Badu-Apraku and Fakorede 2017). The seedling develops haustoria, which penetrates the roots of the maize plant to syphon nutrients and photosynthates (Badu-Apraku and Fakorede 2017). As a result, *Striga* parasitism affects the crop growth, biomass partitioning, and the nutrient status of the maize plant. *Striga* infestation in maize causes chlorotic blotches, scorching, or “firing” of leaves particularly around the margins, wilting of foliage, stunting, spindly stems caused by the preferential allocation of biomass to the roots, and poor grain filling (Menkir *et al.* 2012; Badu-Apraku and Fakorede 2017). These deleterious effects of *Striga* are observed on the host plant even before its emergence from the soil (Parker 2013; Badu-Apraku and Fakorede 2017).

The deleterious impact of *Striga* has been reported in 25 countries in Africa, the most severely affected being sub-Saharan Africa (SSA) countries (Parker 2012). A single *S. hermonthica* plant can inflict an approximately 5% loss in yield on a host plant (Parker and Riches 1993), and high infestation can lead to total crop failure (Badu-Apraku and Fakorede 2017). *Striga* has thus become a major threat to food security, worsening hunger and poverty, especially SSA countries (Pennisi 2010; Khan *et al.* 2014). This impact of *Striga* is further complicated in SSA due to moisture and nutrient stress caused by increased population pressure, short land fallow periods, and minimal use of inorganic fertilizer (De Groote *et al.* 2005). The severity of *Striga* attack increases with the extent of the soil seed bank, existence of strain, variants and races with different virulence, the reaction of the host cultivar, and the environment (Babiker 2007).

*Striga hermonthica* is one of the most difficult parasitic weeds to control (Nickrent and Musselman 2004). The approaches for *Striga* control have been grouped into 4 independent options, which includes cultural, chemical, genetic, that is the use of resistant cultivars and biological control methods (Babiker 2007; Sibhatu 2016). The control measures, such as hand pulling, irrigation, crop rotation, herbicides, fallowing, high level of N application, have proven ineffective in small holder farms (Gressel *et al.* 2004). This is primarily because of the parasite’s highly specialized cycle, which is synchronized with the host’s growth, ability of the parasite to parasitize a broad host range, and longevity of seed of the parasite in the soil (Ejeta 2007; Herne 2009). An integrated management method that involves the use of a number of individual technologies combined together to act at different stages of the parasite's life is sometimes employed for an effective *Striga* control (Kim 1996). However, host plant resistance is the most effective approach against *Striga* damage in maize production and reduces *Striga* seed bank in the soil (Badu-Apraku 2005). This method is the most economical, suitable, and environmentally friendly approach for *Striga* control (Hearne 2009; Sibhatu 2016; Mandumbu *et al.* 2019). Considerable progress in breeding for *Striga* resistance/tolerance have been made by scientists at International Institute of Tropical Agriculture (Badu-Apraku and Lum 2007; Menkir *et al.* 2012; Badu-Apraku, Fakorede *et al.* 2016; Badu-Apraku *et al*., 2016; Yallou *et al.* 2016; Akaogu *et al.* 2019; Menkir and Meseka, 2019).

The advancement in molecular breeding through rapid genotyping and next-generation sequencing technologies has enabled the use of genome-wide association study (GWAS) to be used as a tool for revealing genotype–phenotype associations in crop species (Liu and Yan 2019). GWAS has been found to be a powerful approach for identifying functional genes and alleles that are associated with complex traits in certain environments (Li *et al.* 2016; Zhu *et al.* 2018). This association is based on linkage disequilibrium which is a result of association of a particular trait with a neighboring genetic variation of another trait (Liu and Yan 2019). Unlike quantitative trait locus (QTL) mapping which results in a relatively low-resolution map, genome-wide sequence association mapping gives a relatively high-resolution mapping for identifying genes or regions associated with a particular trait (Xue *et al.* 2013; Liu and Yan 2019). Several studies have employed GWAS to detect QTLs and genomic regions associated with biotic and abiotic stresses (Shikha *et al.* 2021). Wang *et al.* (2012) identified 18 novel candidate genes associated with head smut resistance in maize, 22 QTLs were revealed for gray leaf spot among biparental populations and association mapping panel of 410 tropical/subtropical inbred lines (Kibe *et al.* 2020). Adewale *et al.* (2020) conducted GWAS on *Striga* resistance traits with 132 early maturing inbred lines. Two putative genes (ZmCCD1 and amt5) located on chromosome 9 and 10 were found to

be linked to plant defense mechanism against *Striga*. In another study involving 380 diverse tropical inbred lines, Gowda *et al.* (2021) identified a set of 32 candidate genes physically near the significant SNPs with varying roles in plant defense against biotic stresses. Although some GWAS studies have been conducted to detect candidate genes for *Striga* resistance, none of the QTLs detected have been employed in *Striga* resistance breeding. Therefore, there is a need to conduct additional studies using different genotypes to detect more QTL so as to facilitate the introgression of novel *Striga* resistant genes into maize breeding programs in SSA. The objectives of this research were to (1) determine the genetic structure of a panel of 141 diverse extra-early maturing white quality protein maize (QPM) inbred lines with varying levels of resistance to *S. hermonthica* parasitism and (2) identify significant SNPs and putative genes associated with grain yield and other *Striga* adaptive traits under *Striga*-infested conditions.

## Materials and methods

### Genetic materials

One hundred and sixty-nine extra-early QPM inbred lines from the International Institute of Tropical Agriculture (IITA) Maize improvement program (MIP) were used for this study. The inbred lines comprised 163 S_8_ inbred lines, 4 standard IITA testers (TZEEQI 294, TZEEQI 321, TZEEQI 7, and TZEEQI 134), and 2 inbred checks (TZEEQI 11 and TZEEQI 60) with combined resistance to *Striga*, tolerance to drought and low soil N. The 163 inbred lines were extracted from the F_1_ maize hybrids of 9 biparental crosses involving crosses among extra-early white QPM inbred testers and early maturing white QPM inbred testers. The testers and the checks were extracted from *Striga*-resistant populations. The F1 hybrids were taken through a cycle of backcrossing to the extra-early inbred testers to recover the earliness. The BC_1_F_1_ with desirable agronomic characteristics were selected using the pedigree selection method from each backcrossed population, and advanced through repeated inbreeding to the S_8_ generation.

### Field trials

The experiments were conducted under *Striga*-infested conditions at Mokwa (9° 18′N and 5° 04′E, 457 m asl, 1,100 mm annual rainfall) in 2019 and 2020 and Abuja (9° 15′N and 7° 20′E, 300 m asl, 1,700 mm annual rainfall) in 2020. At all locations, the experiments were laid out using a 13 × 13 lattice design with 2 replicates and single row plots each 3-m long, spaced 0.75 m apart with 0.4 m between plants in each row. Three seeds were sown per hill, and later thinned to 2 plants per hill at 2 weeks after planting (WAP) to obtain a plant population density of 66,000-plants ha^−1^.

Each plot was artificially infested with about 5,000 germinable *S. hermonthica* seeds/hill. The *Striga* infestation method developed by IITA-MIP was adopted to ensure uniform *Striga* infestation with no escapes (Kim 1991; Kim and Winslow 1991). The amount of fertilizer applied was about 30 kg ha^−1^ and was split applied. The time of first application was delayed to 21 days after planting so as to subject the maize plants to stress to stimulate the production of strigolactones in an effort to enhance good germination of *Striga* seeds, and the attachment of the *Striga* plants to the roots of the maize plants. Top dressing was done at about 35 days after planting. Weeds other than *Striga* were constantly removed by hand to ensure good weed control.

### Data collection

Data were collected on the number of emerged *Striga* plants at 8 and 10 WAP and host plant damage syndrome rating at 10 WAP. The host plant damage syndrome rating was recorded on a scale of 1–9 (1 = normal plant growth, no visible symptoms, and 9 = complete scorching of all leaves, causing premature death or collapse of host plant and no ear formation; Kim 1991). Data were also collected on ear aspect, number of ears per plant, and grain yield (Badu-Apraku *et al*. 2011).

### Data analysis

Analysis of variance was performed for the inbreds evaluated in *Striga*-infested environments using the PROC GLM in SAS 2014. The entry means were adjusted for block effects, according to the lattice design. Each year–location combination was considered as a test environment. The environments, replications, and blocks were treated as random factors. Data on the number of emerged *Striga* plants were transformed as [log (counts + 1)] to reduce the heterogeneity of variance for *Striga* counts. Restricted maximum likelihood estimates of the genetic and phenotypic variances of the inbreds were obtained with SAS PROC Varcomp and used to compute the broad-sense heritability for each trait. Correlation analysis was done using the performance analytics package in R. The phenotypic data across environments were collapsed to a single best linear unbiased estimate (BLUE) value using the linear mixed models in META—R (Bates *et al.* 2015; Alvarado *et al.* 2020) as follows:

$$Y_{\mathrm{IJKL}\;}=\;\mu +\;{B(E)}_{J(i)}+\;G_{k}+\;{\mathrm{GE}}_{ij}+\;e_{\mathrm{ijkl}},$$

where *Y_ijkl_* = phenotypic observation for a trait, μ = grand mean, *E* = environmental effect (location), *B*(*E*) = replication effects nested in location, *G* = genotypic effect, GE = genotype by environment interaction, *e* = random residual error. Broad sense heritability (*H*^2^) estimates were calculated from the phenotypic variance (σ2p) and the genotypic variance (σ2g) (Hallauer *et al*. 2010).

### Genotyping and genotypic data analysis

One leaf per plant was collected from 15 representative plants to form a bulk of each of 141 inbred lines 2 WAP in the IITA maize breeding nursery in Ibadan. The leaf tissues were placed in jute bags and freeze-dried using FreeZone Freeze Dryer (Labconco, USA) following the recommendations of the manufacturer's manual. Genomic DNA was isolated from freeze-dried leaf tissues of each inbred line following the modified Cetyl-trimethyl ammonium bromide (CTAB) protocol as described by Azmach *et al.* (2013). The DNA quality and quantity analysis were performed using the UV/Vis Absorbance protocol in the FlUOstar Omega microplate reader (BMG LABTECH) following the manufacturer's manual. Genotyping analysis of the inbred lines was performed using the high-density whole-genome profiling of Diversity Arrays Technology sequencing (DArTseq). The extracted genomic DNA samples were sent to DArT Pty Ltd, Australia (https://www.diversityarrays.com) for DArTseq analysis following the protocol described by Jaccoud *et al.* (2001).

High-throughput genotyping was carried out in 96 plex following the DArTseq protocol. The 49,184 DArTseq markers obtained as raw SNPs were filtered to eliminate SNPs with missing rate greater than 10%, heterozygosity greater than 20%, and minor allele frequency (MAF) less than 5%. SNPs with unknown or multiple chromosome locations were also eliminated. After quality filtering, a total of 8,144 DArTseq markers distributed across the 10 maize chromosomes were retained for the population structure and for GWAS analyses.

### Population structure and kinship analysis

Population structure and kinship analyses were conducted to determine the extent of genetic diversity among the inbred lines. Structure software version 2.3.3 (Pritchard *et al.* 2000) was used to cluster the 141 inbred lines into populations. Structure simulations were carried out using an admixture model with a burning period of 10,000 iterations, followed by Markov chain Monte Carlo set at 10,000. The assumed number of subpopulations was simulated from *k* = 1 to *k* = 10 for an initial assessment of the most likely number of subpopulations, each K was run 10 times. The ideal number of sub-populations (K) was found by examining the optimal ΔK value (Evanno *et al.* 2005) in STRUCTURE Harvester (Earl and von Holdt 2012). Structure population was then plotted using barplot function implemented in R. The phylogeny tree was constructed using ape version 5.0 implemented in R (Paradis and Schliep 2019). The marker-based kinship matrix K was calculated with the same genotypes using the VanRaden method, and then used to create a clustering heat map of the association mapping panel in the GAPIT (Lipka *et al.* 2012).

### Association analysis

The association between SNP genotypes and the phenotypes was determined using a compressed linear model implemented in GAPIT (Genome Association and Prediction Integrated Tool)—R package (Lipka *et al.* 2012). Mixed linear method (MLM) and SUPER (Tang *et al.* 2016) were tested for association analysis. The MLM adopted was proposed by Yu *et al.* (2006) with each molecular marker considered a fixed effect and evaluated individually: *Y* = *Xβ* + *Wα* + *Qv* + *Zu*  + ε; where *Y* is the observed vector of means; *β* is the fixed effect vector (p × 1) other than molecular marker effects and population structure; *α* is the fixed-effect vector of the molecular markers; *ν* is the fixed-effect vector from the population structure; u is the random effect vector from the polygenic background effect; *X*, *W*, and *Z* are the incidence matrice from the associated *β*, *α*, *ν*, and *u* parameters; *ε* is the residual effect vector. Quantile–quantile (Q–Q) plots were generated by plotting the negative logarithms (−log10) of the *P*-values against their expected *P*-values to fit the appropriateness of the GWAS model with the null hypothesis of no association and to determine how well the models accounted for the population structure. The Manhattan plot was generated for visualizing GWAS on the entire genome and zoom mapping was performed on a particular chromosome after identifying a significant SNP marker. The marker effect or SNP contribution was estimated for the significant SNPs using multiple regression analysis using lme4 function implemented in R where the trait was considered as a response variable while the SNP markers above the Bonferroni threshold for the trait was the independent variable. A threshold of –log (p) = 3 was used to declare significant marker–trait associations, which were determined based on the Q–Q plots and distribution of *P*-values for all the measured traits (Gao *et al.* 2016; Sukumaran *et al.* 2018; Mogga *et al.* 2018).

## Results

### Evaluation of phenotypic traits

The combined analysis of variance of the 169 inbred lines (including the 2 checks) across *Striga*-infested environments is presented in Table 1. The results revealed significant (*P* < 0.05) environment (*E*) and genotype mean squares for measured traits. Genotype × environment interactions were not significant (*P* < 0.05) for the number of emerged *Striga* plants at 8WAP and 10 WAP. Broad sense heritability (*h*^2^) estimates on plot mean basis ranged from 46% for *Striga* damage at 10 WAP to 69% for ear aspect. Moderately high broad sense heritability was observed for the measured traits.

**Table 1.** Mean squares of grain yield and other agronomic traits of 169 extra early maturing QPM inbred lines evaluated across *Striga-*infested conditions in Mokwa 2019 and 2020, and Abuja 2020.

Source	DF	Yield, kg/ha	*Striga* damage ratings at 10WAP	Emerged *Striga* plants at 8 WAP	Emerged *Striga* plants at 10 WAP	Ear aspect	Ears per plants
Environment (E)	2	24,506,574**	324.87**	95.80**	62.48**	182.17**	20.11**
Replicate (ENV)	3	7,491,412**	41.38**	1.77**	1.11**	16.17**	1.21**
Block (ENV*Rep)	74	2,071,579**	2.29**	0.11**	0.13**	1.84**	0.19*
Genotype	168	2,178,729**	2.47**	0.12**	0.15**	4.23**	0.19**
Env*Genotype	336	1,041,579*	1.44**	0.08	0.08	1.39**	0.25**
Error	43	865,017	0.78	0.07	0.07	0.84	0.14
Heritability		65	46	48	47	69	—

*, **Significant at 0.05 and 0.01probability levels, respectively.

**Fig. 1.**
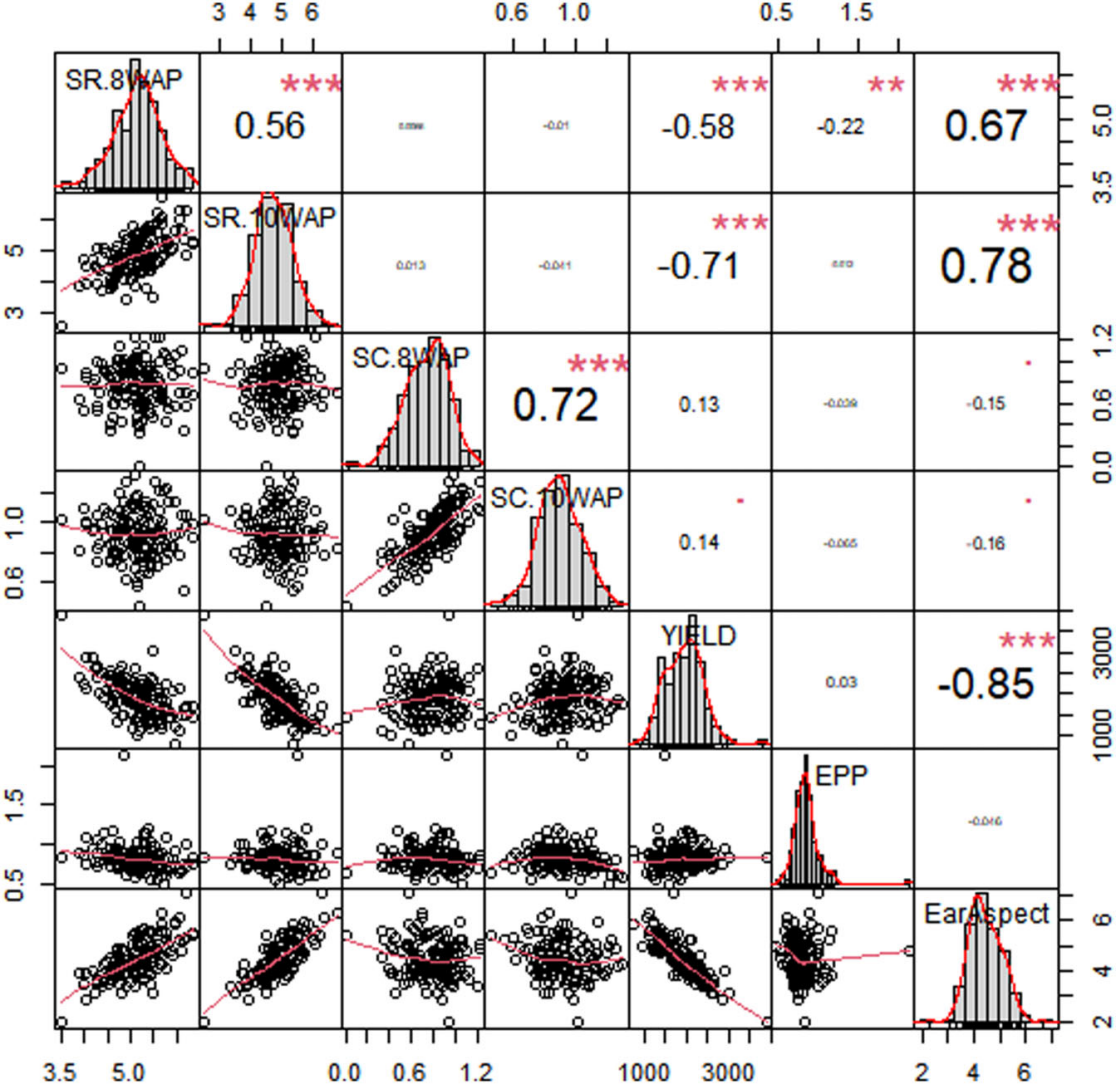
Correlation coefficients between *Striga* resistance indicator traits and other agronomic traits of early maturing QPM inbred lines under artificial *Striga* infestation at Mokwa and Abuja 2019 and 2020. SR 8WAP = *Striga* damage symptoms rating at 8WAP, SR 10WAP = *Striga* damage symptoms rating at 10 WAP, SC 8WAP= number of emerged *Striga* plants at 8 WAP, SC 10WAP = number of emerged *Striga* plants at 10 WAP, YIELD = grain yield, EPP—number of ears per plant, and Ear Aspect = ear aspect.

The phenotypic correlations among grain yield and other measured *Striga* adaptive traits differed under artificial *Striga* infestation (Fig. 1). Grain yield had significant negative correlation with *Striga* damage at 8 WAP (*r* = −0.58**) and 10 WAP (r = − 0.71**), ear aspect (*r* = −0.85**). Significant and positive correlations were obtained between *Striga* damage at 8 WAP and *Striga* damage at 10 WAP (*r* = 0.56**), number of emerged *Striga* plants at 8 WAP and number of emerged *Striga* plants at 10 WAP (*r* = 0.72**), ear aspect and *Striga* damage at 8 WAP (*r* = 0.67**), and *Striga* damage at 10 WAP (*r* = 0.78**).

### Population stratification and genetic diversity

The results revealed that PIC ranged from 0.09 to 0.37 with an average of 0.24 whereas the heterozygosity averaged 0.08 and varied from 0.00 to 0.50 (Supplementary Fig. 1). The mean of the minor allele frequencies of the 8,145 primers was 0.14 with minimum and maximum minor allele frequencies of 0.04 and 0.5, respectively. Gene diversity varied from 0.10 to 0.50 with an average of 0.33.

**Fig. 2.**
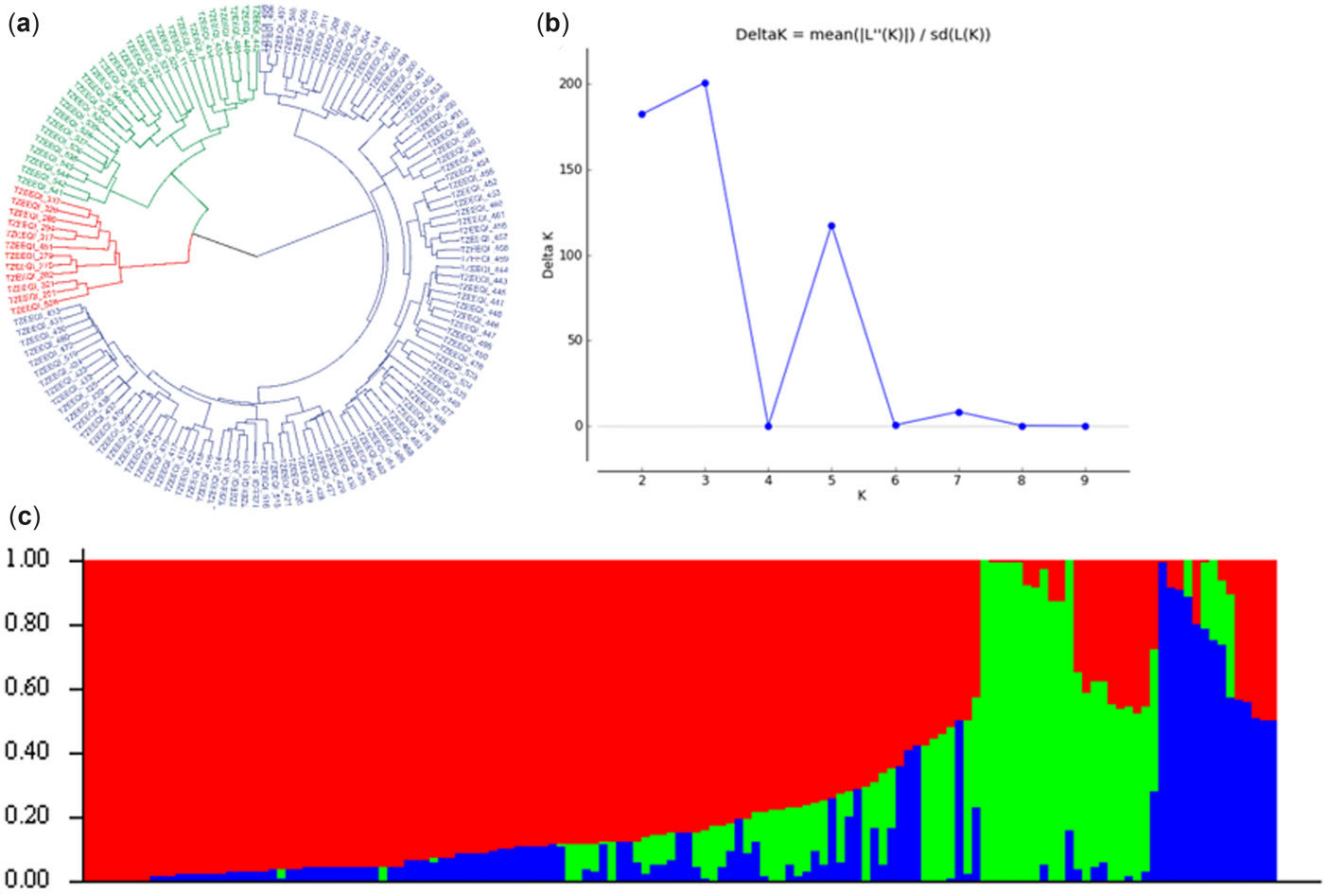
Graphical representation of the population structure of 141 maize inbred lines. a) Phylogeny tree showing the 3 sub-populations. The colors represent each sub-population. b) Plot of mean likelihood of delta K against the number of K groups. The highest peak observed at *K* = 3 signified the grouping of the accessions into 3 groups. c) Population structure originated from the STRUCTURE based on *K* = 3. Each vertical barplot represented a single maize inbred.

The population structure analysis of the inbred lines showed that delta *K* values from the mean log-likelihood probabilities peaked at *k* = 3. At *k* = 3, 84% of the inbred lines were assigned to 3 groups, with only 16% of the lines assigned to the mixed group. A total of 98 inbred lines were placed in group 1, 13 in group 2, 7 in group 3, and 23 in the mixed group. The 3 groups comprised inbred lines derived from 2 or more germplasm sources. The phylogeny tree displayed 3 genetic groups and was aligned with the kinship population structure (Fig. 2). The heat map of the values in the kinship matrix created from the 141 inbred lines also revealed 3 groups which showed relatedness with a few large blocks in the population (Supplementary Fig. 2).

### Genome-wide association analysis

**Fig. 3.**
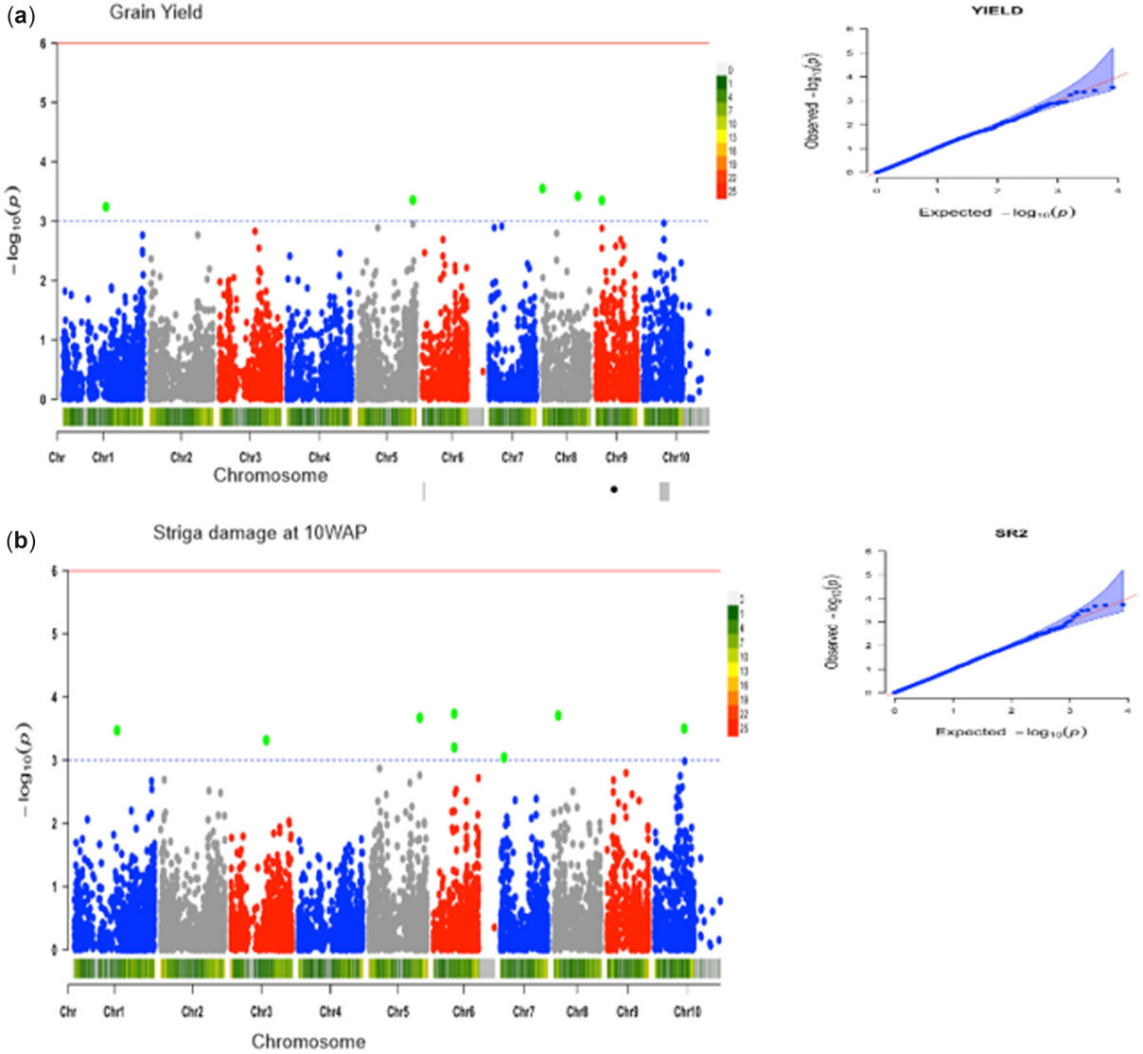
The Manhattan and Q–Q plots of the SNP-based association mapping for (a) grain yield and (b) *Striga* damage at 10 WAP under artificial *Striga* infestation.

**Fig. 4.**
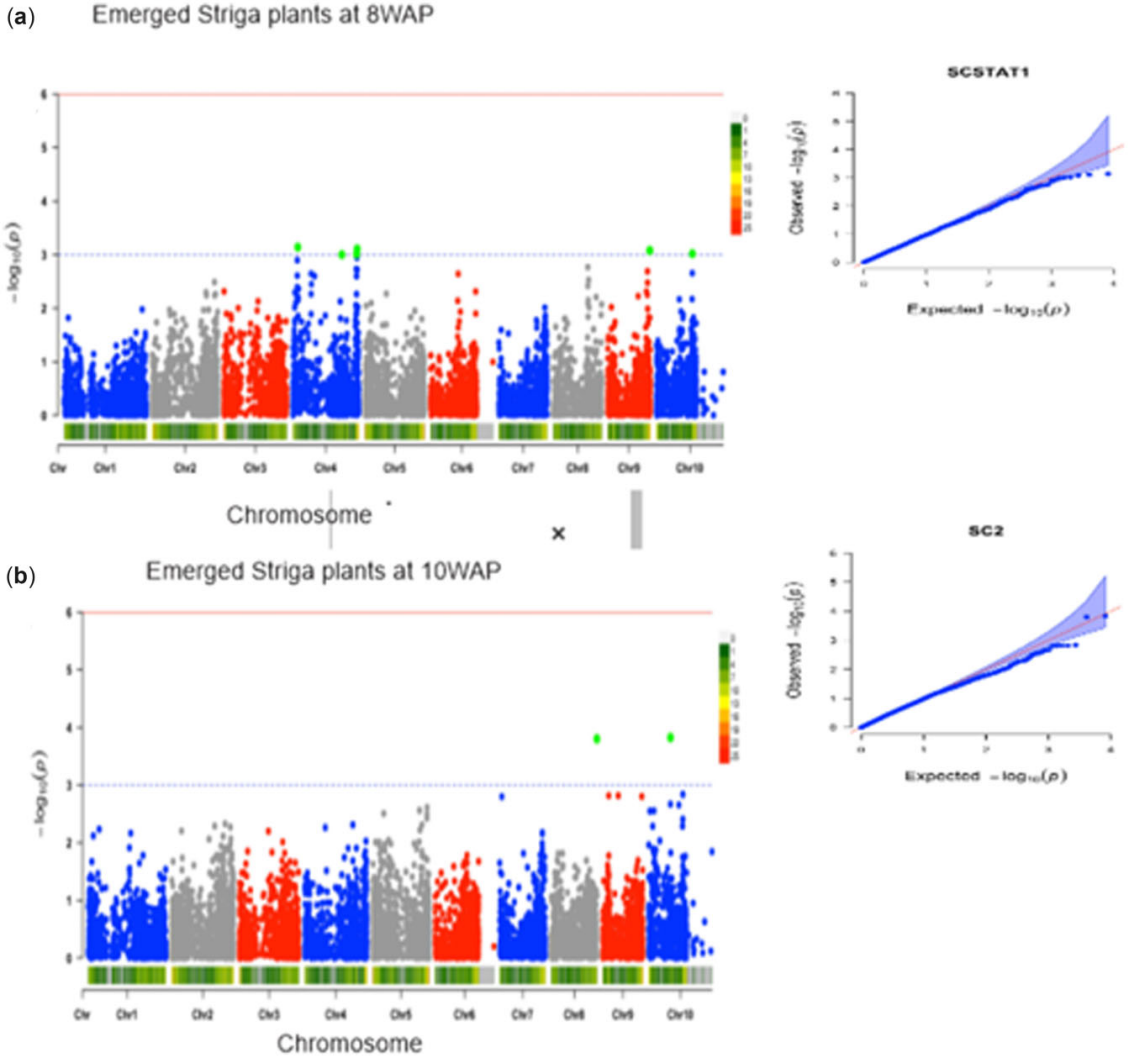
The Manhattan and Q–Q plots of the SNP-based association mapping for (a) number of emerged *Striga* plants at 8 WAP (b) number of emerged *Striga* plants at 10 WAP under artificial *Striga* infestation.

**Fig. 5.**
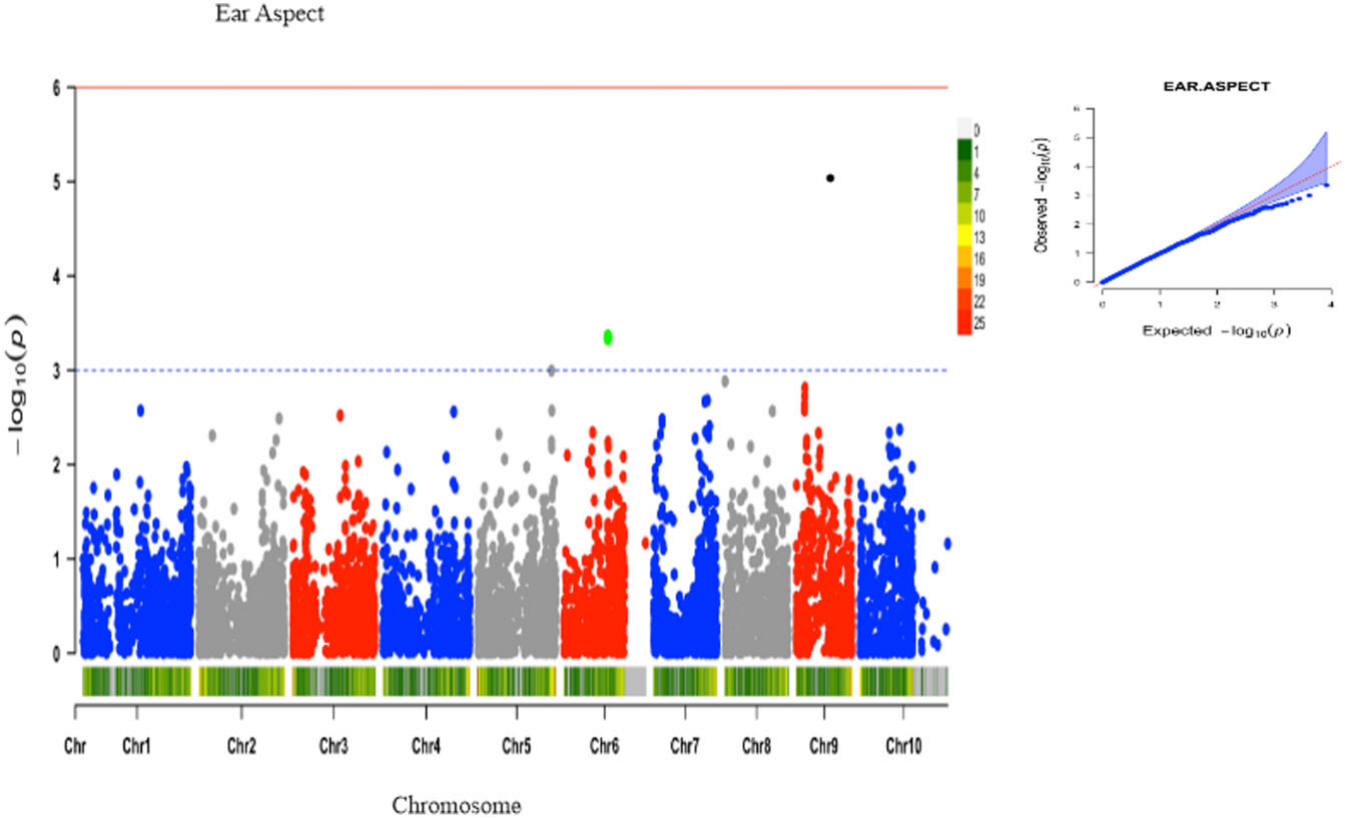
The Manhattan and Q–Q plots of the SNP-based association mapping of ear aspect under artificial *Striga* infestation.

Under artificial *Striga* infestation, a total of 22 significant SNPs were associated with 5 different traits at a GWAS threshold of –log (p) = 3 (Table 2). The trait variation explained by individual marker (*R*^2^) varied from 14% to 22%. Five SNP markers were significantly associated with grain yield (Fig. 3). These markers were located on chromosomes 1, 5, 8, and 9, with the phenotypic variation explained by these markers ranging from 20% to 21%. Four of these markers had negative quantitative trait nucleotide (QTN) effects with the MAF ranging from 0.05 to 0.13. *Striga* damage ratings at 10 WAP was associated with 8 markers (Fig. 3). These markers were located on chromosomes 1, 3, 5, 6, 7, 8, 10, and they explained 14–17% phenotypic variation. Four of the associated markers had negative QTN effects with the MAF ranging from 0.05 to 0.12. Six markers located in chromosomes 4, 9, and 10 were detected for emerged *Striga* plants at 8 WAP (Fig. 4). These markers accounted for 14–15% of the phenotypic variation. Four of these associated markers had negative QTN effects with the MAF ranging from 0.06 to 0.48. Two markers located on chromosomes 8 and 10 were associated with the number of emerged *Striga* plants at 10 WAP (Fig. 4) and explained 21% of the phenotypic variation. One of the SNP markers had MAF varying from 0.13 to 0.48. One SNP marker located on chromosome 8 was associated with ear aspect (Fig. 5) and explained 22% of the phenotypic variation. The MAF of this marker was 0.50. Marker S1_163520946 located on chromosome 1 was repeatedly found to be associated with grain yield and *Striga* damage ratings at 10 WAP.

**Table 2.** Summary of SNP markers associated with *Striga*-adaptive traits evaluated under *Striga*-infested conditions.

Traits	SNP maker	Chr	Position	MAF	-log10(P)'	*P*-value	*r* ^2^ (%)	Effect
Grain yield	chr8_328928	8	328928	0.13	3.55	2.82 × 10^−4^	21.38	−390.03
	chr8_135475183	8	135475183	0.07	3.42	3.80 × 10^−4^	20.99	−325.99
	chr5_210580022	5	210580022	0.12	3.36	4.41 × 10^−4^	20.79	−409.87
	chr9_23155189	9	23155189	0.10	3.35	4.47 × 10^−4^	20.77	254.16
	chr1_163520946	1	163520946	0.05	3.24	5.76 × 10^−4^	20.44	−334.13
*Striga* damage ratings at 10 WAP	chr6_79398477	6	79398477	0.09	3.73	1.8 × 10^−4^	17.17	0.48
	chr8_17232945	8	17232945	0.48	3.71	1.96 × 10^−4^	17.08	−0.24
	chr5_194107033	5	194107033	0.09	3.67	2.14 × 10^−4^	16.96	−0.68
	chr10_112661466	10	112661466	0.12	3.50	3.17 × 10^−4^	16.41	0.38
	chr1_163520946	1	163520946	0.05	3.47	3.38 × 10^−4^	16.32	0.51
	chr3_135777833	3	135777833	0.06	3.31	4.85- × 10^−4^	15.81	−0.46
	chr6_79656637	6	79656637	0.07	3.20	6.30 × 10^−4^	15.45	−0.44
	chr7_14310701	7	14310701	0.07	3.04	9.07 × 10^−4^	14.95	0.42
Emerged *Striga* plants at 8WAP	chr4_18563948	4	18563948	0.20	3.14	7.22 × 10^−4^	16.16	−0.08
	chr4_240388223	4	240388223	0.13	3.11	7.80 × 10^−4^	16.06	0.10
	chr9_156894729	9	156894729	0.06	3.08	8.31 × 10^−4^	15.97	0.19
	chr10_134476659	10	134476659	0.07	3.02	9.57 × 10^−4^	15.78	−0.12
	chr4_238158257	4	238158257	0.17	3.01	9.74 × 10^−4^	15.75	−0.09
	chr4_183275539	4	183275539	0.10	3.00	9.96 × 10^−4^	15.72	−0.15
Emerged *Striga* plants at 10WAP	chr10_85787634	10	85787634	0.48	3.83	1.50 × 10^−4^	21.86	1.65
	chr8_180192054	8	180192054	0.13	3.80	1.58 × 10^−4^	21.79	−2.99
Ear aspect	chr6_125302216	6	125302216	0.50	3.35	4.49 × 10^−4^	22.38	0.23

## Discussion

### Phenotypic variation

The significant genotypic variation observed among the inbred lines for grain yield and other measured traits in our study indicated the existence of genetic variability among the extra-early maturing QPM inbred lines. The significant environmental effects for measured traits indicated that the research conditions were unique and provided distinct information on the QPM inbred lines. The differential response of the genotypes for grain yield and other measured traits suggested that the inbred lines performed differently under the research condition. This differential performance could be attributed to variation in environmental factors such as temperature, rainfall, and soil types in the different test environments (Badu-Apraku and Lum 2007; Badu-Apraku *et al.* 2010).

The moderate to high heritability estimates observed for grain yield and other *Striga* adaptive traits implied the efficiency of SNP in the maize panel in the identification of true associations between the markers and putative genes (Adewale *et al.* 2020). The inverse relationship between grain yield and number of emerged *Striga* plants and *Striga* damage syndrome ratings implied that increased number of emerged *Striga* plants led to a lower grain yield (Menkir *et al.* 2012) and that lower *Striga* damage syndrome ratings led to an increased grain yield (Gowda *et al.* 2021). Previous studies recommended the simultaneous selection of reduced *Striga* damage syndrome ratings and the number of emerged *Striga* plants as indicators for *Striga* resistance under field infestation (Menkir and Kling 2007; Kim 1994; Kling *et al.* 2000).

### Genome-wide association studies

In the GWAS analysis, the population structure information was used to correct possible false discovery. Q–Q plots were generated by comparing the observed and expected *P*-values under the null hypothesis of no associations to determine how well the models accounted for the population structure. The results revealed that majority of points in the Q–Q plots were aligned on the diagonal line for all the measured traits indicating that the model successfully accounted for population structure and familiar relationships in the GWAS analysis. The whole-genome scan for phenotypic and allelic variation in Maize *Striga* resistance identified 9 genomic regions on chromosomes 10, 9, 8, 7, 5, 4, 3, and 1 with significant −log10 values. At a threshold of –log (p) = 3, a total of 22 markers were identified to be significantly associated with *Striga* damage, number of emerged *Striga* plants, ear aspect, and grain yield under *Striga* infestation. Information on the SNP markers from this study could accelerate the use of genomics-informed selection techniques in breeding *Striga* resistant maize cultivars.

Several QTL associated with various *Striga* resistance indicator traits have been reported in earlier studies. For example, Badu-Apraku *et al.* (2020) identified 116 QTLs associated with 4 *Striga* resistance traits using biparental population derived from 2 early maturing maize lines. A similar study by Badu-Apraku *et al.* (2020) revealed 14 QTLs associated with 3 *Striga* resistance traits. Adewale *et al.* (2020) reported 24 markers significantly associated with *Striga* damage, number of emerged *Striga* plants, number of ears per plant, ear aspect, and grain yield. Also, Gowda *et al.* (2021) identified a total of 57 significant markers for *Striga* resistant traits and yield distributed across the maize genome and controlled by a few major and many minor genes. Stanley *et al.* (2021) identified 30 significant SNPs that were significantly associated with 3 *Striga* resistance trait. Among these studies, S8_17232945 which was detected for *Striga* damage syndrome rating in this study was found to overlap with 1 QTL reported by Badu-Apraku *et al.* (2020). The differential QTL mappings observed in these experiments could be attributed to differences in the genetic materials used for the studies (Kaur *et al.* 2021).

The QTL analysis in this study, and previous studies provided information on the chromosonal regions controlling *Striga* resistance, which can be crucial to breeding *Striga* resistance cultivars through marker-assisted breeding. However, these QTLs have not been utilized in maize *Striga* resistance breeding due to factors, such as limited marker–trait association, a low number of markers used in mapping, small phenotypic variance explained, differences in the genetic backgrounds, and environmental effects (William *et al.* 2007; Tuberosa 2012). A meta-QTL analysis of the results of these findings can be employed to refine the number and position of the QTLs to identify stable QTLs (Sheoran *et al.* 2022). Meta-QTL analysis has been conducted to successfully locate the regions in the genomes of various traits in different crops. In maize, Chen *et al.* (2017) subjected 999 QTLs to meta-QTL analysis and obtained a total of 76 MQTLs across the maize genome. Three potential candidate genes (GRMZM2G359974, GRMZM2G301884, and GRMZM2G083894) were associated with kernel size and weight within 3 MQTL using regional association mapping. Guo *et al.* (2018) using a total of 428 individual QTLs for 23 root-related traits identified 53 Meta-QTLs (MQTLs) retrieved over 10 maize chromosomes. Three maize genes (GRMZM5G813206, GRMZM2G167220, and GRMZM2G467069) that could play important roles on lateral root and crown root development of maize were also identified. Wang *et al.* (2022) carried out MQTL analysis using 282 QTLs from 25 experiments and identified 11 and 34 MQTLs associated with grain dry matter and low grain water content, respectively. Sheoran *et al.* (2022) using a total of 542 QTLs, detected 32 mfeta-QTL possessing 1,907 candidate genes for different abiotic (drought, water logging, heat, and cold) stresses. Furthermore, MQTL 2.1, 5.1, 5.2, 5.6, 7.1, 9.1, and 9.2 were found to control different stress-related traits for combined abiotic stress tolerance. Meta-QTL analyses have also been conducted in crops such as Barley (Zhang *et al.* 2017; Khahani *et al.* 2019), Wheat (Soriano *et al*. 2021; Venske *et al.* 2019), Rice (Sandhu *et al.* 2021; Prakash *et al.* 2022), and Cotton (Said *et al.* 2013; Xu *et al.* 2020).

## Conclusion

The 141 QPM inbred lines used for this study displayed high genetic variability in response to the *Striga*-related traits. The GWASs conducted in this research revealed 22 SNPs that were significantly associated with *Striga* resistance adaptive traits. The identified SNP markers after validation would be invaluable for molecular breeding for maize *Striga* resistance in SSA. A meta-QTL analysis should be employed for the identification of stable QTLs from existing genomic regions that have been associated with yield and other *Striga* resistance traits. This will facilitate the detection of putative genes underlying *Striga* resistance in maize.

## Supplementary Material

jkac237_Supplementary_Data

## Data Availability

The datasets used in the present study are available at the IITA CKAN repository. Genotypic data of 141 extra-early maturing quality protein maize inbred lines: https://doi.org/10.25502/k6f5-t130/d. Phenotypic data of 169 extra-early maturing quality protein maize evaluated under *Striga* conditions across 3 environments, 2019–2020: https://doi.org/10.25502/14yp-5w46/d. Supplemental material is available at G3 online.
