# Supplementary material for: Genome-wide association studies of Striga resistance in extra-early maturing quality protein maize inbred lines
Source: G3 (Bethesda). 2022 Sep 8;13(2):jkac237. doi: 10.1093/g3journal/jkac237 (PMC9911053; doi:10.1093/g3journal/jkac237)
Supplement: jkac237_Supplementary_Data [file jkac237_supplementary_data.docx]

**Supplementary Files**

**
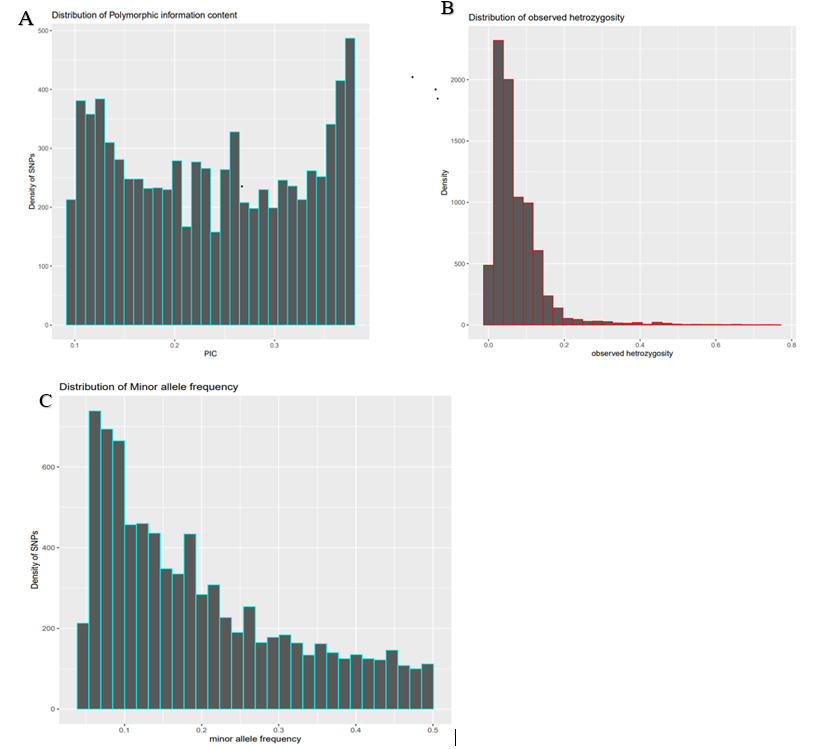
**

**Figure S1: (a) Polymorphic information content of 8143 SNPs markers. (b) Observed heterozygosity among 8143 SNPs markers. (c) Distribution of minor allele frequency**

**
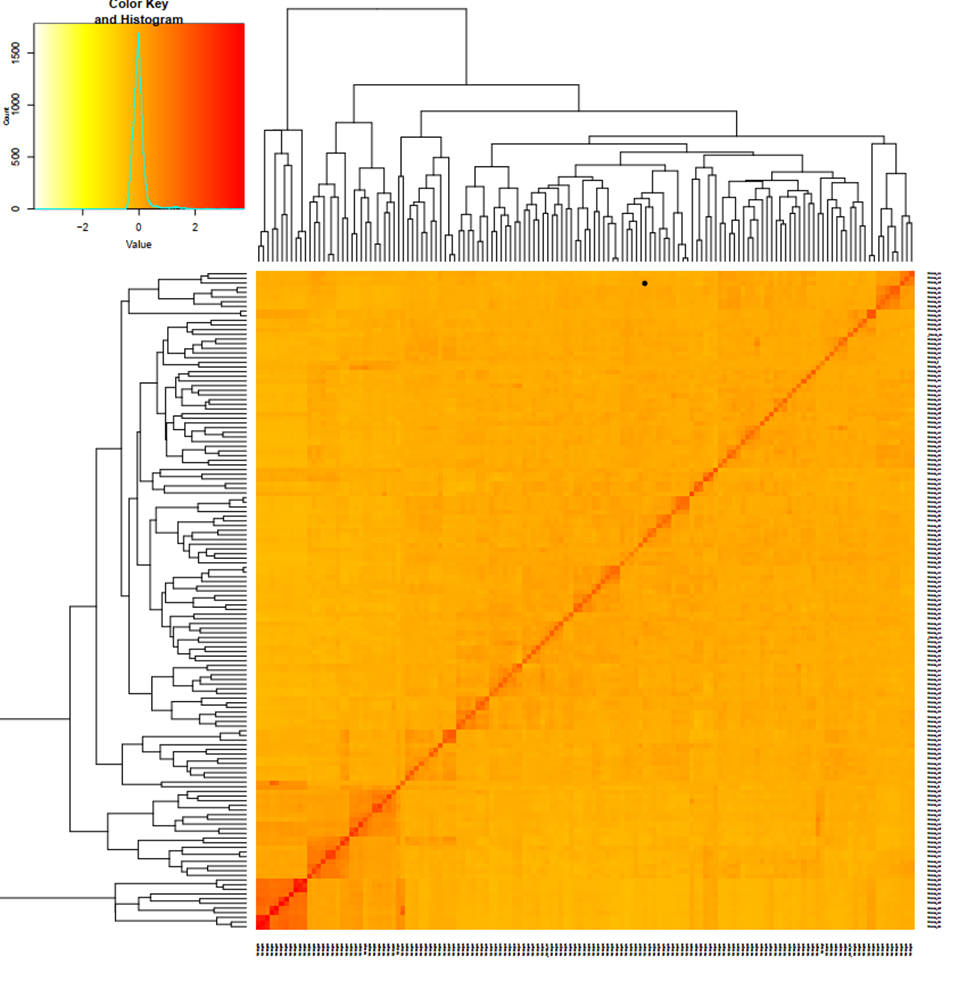
**

**Figure S2**. **Heat map showing the kinship analysis. The kinship values showed a normal distribution (turquoise curve), and orange and red colors represent weak and high kinship relations in the panel, respectively. The resulted clustering tree is indicated outside of the matrix.**
